# Supplementary material for: Assessment of the Toxic Metals Pollution of Soil and Sediment in Zarafshon Valley, Northwest Tajikistan (Part II)
Source: Toxics. 2020 Nov 23;8(4):113. doi: 10.3390/toxics8040113 (PMC7712287; doi:10.3390/toxics8040113)
Supplement: Supplementary file 1 [file toxics-08-00113-s001.pdf]

Supplementary Materials: Assessment of the Toxic Metals Pollution of Soil and Sediment in Zarafshon Valley, Northwest Tajikistan (Part II)

Daler Abdusamadzoda, Djamshed A. Abdushukurov, Octavian G. Duliu and Inga Zinicovscaia

Table S1. The values of contamination factor of soils (i) and sediments (h). Anthropogenic exposed soils and sediments are marked in red.

Sampling Sites	CF(V)	CF(Cr)	CF(Mn)	CF(Co)	CF(Ni)	CF(Zn)	CF(As)	CF(Sb)	CF(Ba)	CF(W)	CF(Hg)	PLI
						Soils						
i-01	0.61	0.43	0.64	0.46	0.67	0.88	4.71	9.00	0.38	1.09	1.22	1.00
i-02	0.97	1.01	0.61	0.66	0.96	1.43	5.13	54.00	0.87	1.42	5.86	1.89
i-03	0.8	0.7	0.7	0.5	1.2	1.3	3.8	35.5	0.8	1.1	6.8	1.6
i-04	0.9	0.7	0.7	0.3	1.3	1.0	5.5	23.8	0.7	0.8	5.4	1.5
i-05	0.7	0.8	0.6	0.4	1.0	1.4	4.4	29.5	0.6	0.7	4.2	1.5
i-06	1.39	0.88	0.69	0.55	2.49	1.63	7.33	727	0.82	0.95	128	3.51
i-07	1.07	1.17	0.76	0.63	1.94	1.84	6.08	1595	1.04	1.26	876	4.68
i-08	0.2	0.2	0.5	0.6	1.1	1.4	0.9	2.8	0.8	0.9	6.0	0.9
i-09	0.6	0.4	0.6	0.7	1.4	1.6	3.3	3.8	0.5	1.2	2.0	1.1
i-10	2.3	1.0	0.8	0.8	1.6	1.9	5.6	13.3	0.7	1.8	3.3	1.9
i-11	1.9	1.1	0.8	1.2	1.4	1.7	3.9	14.5	0.6	1.9	2.6	1.8
i-12	1.1	0.7	0.7	0.9	0.9	1.0	2.8	10.8	0.8	1.2	1.1	1.3
i-13	1.7	1.2	0.6	1.2	1.3	1.4	4.6	12.0	0.6	1.8	6.0	1.9
i-14	1.2	1.1	0.7	1.3	1.4	2.1	5.8	59.3	0.9	2.5	10.0	2.5
i-15	1.52	1.01	0.81	1.01	1.04	1.85	8.44	200	1.26	1.84	26.00	3.09
i-16	2.43	1.21	0.92	1.09	1.25	1.58	6.73	44.25	1.22	2.89	74.00	3.27
i-17	2.82	1.13	0.83	0.91	1.72	1.76	8.29	31.25	1.31	3.05	50.00	3.21
i-18	1.14	1.99	0.61	1.39	3.11	1.09	1.92	4.05	0.58	1.03	1.20	1.39
i-19	0.62	0.72	0.58	0.49	0.89	0.75	5.15	24.50	0.54	1.03	0.76	1.15
i-20	0.74	0.82	0.70	0.62	1.09	1.16	50.00	140	0.69	2.95	1.24	2.20
i-21	0.90	4.53	0.50	0.42	4.06	3.78	1237	6400	0.93	13.32	28.00	9.12
i-22	0.5	0.2	0.3	0.4	0.4	0.5	0.7	4.3	0.4	1.8	34.0	0.9
i-23	0.8	0.4	0.5	0.5	0.6	0.7	1.5	7.8	0.6	1.4	24.0	1.2
i-24	1.1	0.6	0.6	0.6	0.7	0.9	2.9	27.5	0.7	1.0	2.0	1.3
i-25	1.6	0.9	0.7	1.1	1.2	1.3	6.7	22.3	1.0	1.9	44.0	2.5
i-26	1.2	0.8	1.0	0.8	1.0	1.4	3.2	8.9	0.6	1.1	5.0	1.5
i-27	1.0	0.9	1.0	0.8	1.1	1.1	3.1	7.3	0.6	1.0	4.5	1.4
i-28	0.4	0.6	0.5	0.3	1.3	0.7	1.9	0.5	0.2	0.7	2.0	0.7
i-29	0.5	0.6	0.5	0.4	1.1	0.5	1.4	0.8	0.2	0.4	12.0	0.7
i-30	0.3	0.3	0.5	0.4	1.1	0.8	2.2	0.5	0.3	0.2	6.0	0.6
i-31	0.3	0.3	0.5	0.5	0.5	0.9	1.8	1.3	0.4	0.4	4.0	0.7
i-32	0.5	0.3	0.4	0.6	0.8	1.0	3.4	2.8	0.3	0.5	4.0	0.8
i-33	0.9	0.4	0.6	0.8	0.7	1.2	5.8	4.3	0.4	0.7	12.0	1.2
i-34	0.9	0.5	0.5	0.5	1.0	2.0	3.8	8.5	0.4	0.8	4.3	1.3
i-35	1.4	0.6	0.7	1.0	0.7	1.5	7.4	6.8	0.6	1.2	16.0	1.7
i-36	2.9	1.3	1.0	1.6	1.8	2.7	5.0	14.3	1.6	2.3	74.0	3.3
i-37	1.9	1.0	0.9	1.2	2.0	2.0	7.0	11.3	1.5	1.8	70.0	2.9
i-38	1.8	0.8	0.9	0.9	1.6	1.9	10.9	10.3	1.0	1.4	46.0	2.5
i-39	1.32	1.21	0.89	0.94	1.34	1.67	17.08	19.75	0.78	1.53	5.30	2.21
i-40	1.61	1.93	1.08	1.65	1.74	2.46	60.21	34.25	1.24	1.58	50.00	4.05
i-41	1.23	1.01	0.85	0.92	1.36	1.10	15.83	23.25	0.81	2.21	1.26	1.91
i-42	2.89	0.89	1.90	1.94	2.23	3.03	20.63	41.25	0.91	1.58	1.42	2.88
i-43	0.58	0.54	0.44	0.51	0.95	0.61	3.00	9.65	0.42	2.37	0.98	1.01
i-44	1.54	1.29	0.98	1.21	1.96	1.60	7.50	11.50	0.90	2.00	1.22	1.93
i-45	1.39	0.99	0.80	1.16	2.19	1.27	12.19	9.00	0.50	3.89	6.00	2.15
i-46	1.0	0.8	0.7	0.6	1.2	1.2	13.9	18.3	0.5	1.4	56.0	2.2
i-47	1.2	0.9	0.7	0.8	1.5	1.4	12.8	21.5	0.7	0.9	72.0	2.4
i-48	1.3	1.2	1.0	1.1	1.7	1.3	50.0	28.5	1.1	3.7	0.9	2.5
i-49	1.0	1.0	1.1	0.9	1.6	1.7	30.0	21.5	0.8	2.0	1.6	2.1
i-50	1.3	0.8	0.8	0.7	1.4	2.1	6.5	16.5	1.0	1.1	0.8	1.6
i-51	1.2	1.0	0.8	1.0	1.4	1.4	3.2	12.5	1.0	1.3	1.3	1.6
i-52	1.4	0.8	0.9	0.9	1.8	1.6	15.9	23.3	1.0	2.0	48.0	2.8
i-53	0.1	0.3	0.7	0.4	0.6	1.5	0.8	3.5	1.3	1.1	10.0	0.9

i-54	0.2	0.4	0.8	0.4	0.9	1.6	0.5	3.3	1.5	1.2	10.0	1.0
i-55	0.2	0.4	0.8	0.5	1.2	1.8	0.6	8.3	1.5	0.9	12.0	1.2
i-56	1.0	0.5	0.9	0.7	1.3	2.0	0.9	6.5	1.6	1.2	14.0	1.5
i-57	1.1	0.8	1.0	0.8	1.4	2.6	1.4	9.3	1.7	1.5	12.0	1.9
i-58	1.5	1.0	1.1	1.0	1.5	1.6	1.6	6.0	2.0	1.0	16.0	1.9
i-59	1.8	1.2	1.1	1.2	1.7	2.4	2.1	9.5	2.1	1.5	26.0	2.4
i-60	1.7	1.1	1.1	1.1	1.0	2.1	5.0	8.4	3.5	1.2	4.6	2.1
i-61	1.9	1.3	1.1	1.3	1.9	1.9	1.9	11.0	2.6	1.3	14.0	2.4
i-62	1.2	1.0	1.1	0.8	0.8	2.1	3.0	7.9	2.4	0.9	3.9	1.7
i-63	1.5	1.1	1.2	1.1	1.1	2.6	2.5	6.3	2.3	1.1	4.3	1.9
i-64	1.2	0.9	0.9	0.8	0.8	1.8	3.1	6.6	2.0	0.9	4.0	1.6
i-65	1.7	1.1	1.2	1.0	1.1	2.2	4.4	7.3	2.2	1.0	5.5	2.0
i-66	1.4	1.3	1.3	1.2	1.5	2.6	1.2	6.0	2.4	1.2	12.0	2.0
i-67	1.9	1.1	1.3	1.3	1.4	1.1	1.8	4.8	2.2	1.2	8.0	1.9
i-68	1.5	1.0	1.0	1.3	1.1	2.2	3.7	8.3	2.0	1.1	5.8	2.0
i-69	1.5	1.2	1.1	1.0	1.1	2.4	5.4	8.3	1.9	1.0	6.2	2.1
i-70	2.3	1.3	1.4	1.4	1.9	2.8	4.0	6.5	2.2	1.4	14.0	2.6
i-71	2.1	1.1	1.2	1.1	1.1	2.0	2.6	3.8	2.3	1.2	16.0	2.1
i-72	2.4	1.2	1.1	1.0	1.1	1.8	1.0	3.8	2.4	1.3	14.0	1.9
i-73	2.2	0.9	1.1	0.9	1.3	2.2	2.8	6.3	2.6	1.4	12.0	2.1
i-74	1.8	1.0	1.1	0.7	0.8	1.6	1.6	4.5	1.8	0.9	24.0	1.8
i-75	2.0	1.1	1.0	1.0	0.7	1.7	0.9	6.3	2.0	1.0	22.0	1.8
i-76	3.0	1.2	1.0	1.2	1.5	2.8	1.7	14.3	2.6	1.3	24.0	2.6
i-77	1.59	1.20	1.68	2.88	2.62	3.43	7.29	19.75	1.42	3.00	1.46	2.77
i-78	1.97	0.93	1.45	1.59	1.94	2.40	12.71	41.75	1.42	1.61	1.58	2.61
i-79	149.48	0.97	0.92	1.12	1.38	1.75	5.08	14.75	1.70	1.53	1.40	2.84
i-80	1.1	0.6	0.3	1.0	1.3	2.8	1.6	7.4	1.1	1.1	1.1	1.2
i-81	2.2	0.7	0.5	0.6	1.1	2.9	1.9	9.5	1.7	1.2	10.0	1.8
i-82	2.5	0.8	0.6	0.6	0.9	2.3	1.1	11.3	1.8	1.3	8.0	1.7
i-83	2.3	0.8	0.6	0.6	0.9	1.7	1.1	6.8	1.9	1.0	10.0	1.5
i-84	2.5	0.8	0.9	0.7	0.9	1.5	0.7	9.0	2.2	0.9	8.0	1.6
i-85	1.1	0.7	0.8	0.8	1.0	1.8	4.0	15.8	0.8	1.4	2.5	1.6
i-86	0.6	0.8	0.8	0.5	0.7	1.3	0.8	12.0	1.6	0.7	2.0	1.1
i-87	1.3	1.0	0.8	0.8	1.2	1.5	2.2	6.8	3.3	1.3	1.2	1.6
i-88	2.8	1.0	0.9	0.7	1.1	2.7	2.4	8.3	2.5	1.3	8.0	2.0
i-89	1.6	0.9	0.9	1.0	1.3	2.3	2.8	10.5	2.7	1.9	10.0	2.2
i-90	0.5	0.5	0.7	0.5	0.7	1.3	9.0	4.9	0.5	0.8	0.9	1.0
i-91	1.7	0.7	0.7	0.4	0.5	1.7	5.4	6.5	0.7	0.9	2.0	1.2
i-92	1.4	0.8	0.7	0.4	0.8	1.4	6.5	4.3	1.1	0.6	2.0	1.3
i-93	1.9	1.0	0.9	1.0	1.6	2.6	5.1	7.3	2.0	0.9	8.0	2.1
i-94	1.3	1.3	0.5	0.9	0.8	1.8	0.6	4.4	0.8	1.1	2.9	1.2
i-95	1.3	1.8	0.6	1.1	2.6	1.3	2.4	9.2	0.8	1.1	1.3	1.6
i-96	0.8	0.9	0.4	0.6	1.6	1.0	5.0	23.3	0.5	0.8	2.0	1.3
i-97	1.7	1.9	0.5	0.8	1.9	1.4	3.7	21.0	0.9	1.3	14.0	2.1
i-98	1.9	2.1	0.6	0.7	2.5	1.1	3.0	47.0	0.6	0.9	10.0	2.1
i-99	1.0	0.9	0.5	0.9	1.7	1.7	2.2	18.0	0.9	1.3	8.0	1.7
i-100	0.9	0.8	0.6	0.7	1.4	1.3	1.2	12.3	1.0	1.0	10.0	1.5
i-101	0.5	0.5	0.5	1.0	1.1	1.4	1.3	5.3	0.9	0.6	10.0	1.2
i-102	0.5	0.8	0.4	1.2	1.2	1.6	2.7	6.3	0.8	0.7	26.0	1.5
i-103	0.94	1.75	1.00	0.76	1.96	2.07	15.00	13.25	1.21	0.89	70.00	2.78
i-104	0.1	0.0	0.5	0.1	1.7	0.8	0.4	3.0	0.6	0.3	10.0	0.5
i-105	0.3	0.2	0.6	0.3	2.1	1.1	1.2	6.3	0.7	0.3	26.0	1.0
i-106	0.8	0.7	0.7	0.7	2.5	1.7	4.6	25.5	0.9	1.0	38.0	2.2
i-107	1.2	1.2	0.8	0.4	2.0	1.6	9.0	56.3	0.7	1.4	8.0	2.2
i-108	1.71	2.45	1.15	0.79	1.40	3.85	58.75	349.25	0.79	1.95	10.00	4.16
i-109	2.5	1.4	1.0	0.8	1.9	3.2	4.7	28.8	2.0	1.9	12.0	2.9
i-110	2.96	2.74	0.85	1.61	2.79	4.37	27.29	64.25	1.89	2.47	46.00	5.02
i-111	1.9	2.3	0.9	0.9	2.5	3.7	13.0	19.3	2.2	1.2	24.0	3.3
i-112	1.3	1.4	1.0	0.8	1.1	3.2	6.9	14.3	2.1	1.2	14.0	2.4
i-113	1.2	1.1	1.1	0.9	1.3	2.3	5.6	11.0	2.3	0.9	10.0	2.2
i-114	1.6	0.9	0.9	0.7	1.2	1.5	4.1	16.3	1.9	1.9	16.0	2.2
i-115	0.7	0.7	0.6	0.5	0.7	0.9	2.8	54.3	1.4	3.9	1.0	1.5
i-116	0.9	0.7	0.7	0.6	0.9	1.8	2.6	26.8	1.7	2.5	4.0	1.8
Sediments												
h-01	0.8	0.9	0.4	0.6	0.7	0.7	2.1	6.6	1.2	1.6	5.9	1.29
h-02	0.2	0.2	0.3	0.4	0.5	0.3	2.6	3.4	0.4	1.1	2.2	0.65
h-03	0.7	0.9	0.3	0.6	0.7	0.9	1.3	10.1	1.4	1.5	6.5	1.26
h-04	0.71	0.93	0.33	0.65	0.74	0.98	0.91	16.14	1.42	1.94	18.31	1.49
h-05	0.76	0.98	0.31	0.57	0.98	0.69	0.73	22.21	1.38	2.29	22.99	1.53

h-06	0.74	1.02	0.25	0.83	1.23	1.01	0.65	37.71	1.46	2.42	45.97	1.83
h-07	1.0	1.2	0.3	1.4	1.6	1.1	1.4	127	1.6	3.1	471	3.16
h-08	0.8	1.1	0.3	0.9	2.1	1.2	0.1	3935	1.6	3.9	1818	3.98
h-09	0.36	0.41	0.42	0.28	0.48	0.53	0.52	1.64	1.22	0.50	6.49	0.68
h-10	0.33	0.48	0.44	0.35	0.61	0.56	0.81	3.00	1.02	0.79	6.49	0.81
h-11	0.78	0.74	0.47	0.66	0.68	0.78	1.50	10.43	1.07	1.45	6.49	1.28
h-12	0.73	0.80	0.45	0.57	0.58	0.71	1.59	7.36	1.03	1.29	9.09	1.22
h-13	1.12	1.16	0.50	1.03	1.04	1.06	3.93	3.71	1.23	2.21	18.96	1.78
h-14	0.86	0.98	0.37	0.64	0.76	0.79	2.31	4.86	1.17	2.00	9.09	1.36
h-15	0.76	0.87	0.43	0.54	0.83	0.52	1.94	5.14	0.99	1.79	10.39	1.25
h-16	0.7	0.8	0.4	0.7	0.7	0.6	1.6	6.3	1.0	1.3	3.1	1.10
h-17	0.8	0.9	0.5	0.6	0.7	0.5	1.5	9.3	1.0	1.4	2.9	1.13
h-18	1.1	0.8	0.5	0.6	1.3	0.6	2.1	17.9	1.1	2.6	23.4	1.77
h-19	0.8	0.8	0.7	0.8	0.7	0.9	0.9	0.5	1.6	0.9	5.6	0.95
h-20	0.8	0.6	0.6	0.7	1.0	0.7	1.3	1.0	1.3	1.0	13.0	1.10
h-21	0.6	0.8	0.4	0.5	0.7	0.5	3.6	1.2	1.2	1.3	26.6	1.18
h-22	0.5	1.4	0.2	0.4	0.7	1.6	240	360.7	0.5	20.0	122.1	4.16
h-23	0.26	0.16	0.14	0.12	0.17	0.14	0.63	0.43	0.39	0.71	12.99	0.37
h-24	0.40	0.32	0.20	0.27	0.30	0.22	0.80	0.57	0.43	0.86	15.58	0.54
h-25	0.43	0.42	0.30	0.37	0.42	0.38	1.19	1.48	0.74	0.86	20.39	0.79
h-26	0.88	0.72	0.35	0.48	0.23	0.32	1.79	8.64	0.98	0.71	19.48	1.04
h-27	0.41	0.41	0.19	0.24	0.50	0.66	0.40	1.09	0.44	0.72	49.35	0.69
h-28	0.28	0.38	0.12	0.17	0.28	0.17	0.32	1.04	0.16	0.77	8.05	0.39
h-29	0.19	0.29	0.12	0.21	0.26	0.18	0.29	0.57	0.19	0.36	5.19	0.32
h-30	0.22	0.23	0.11	0.18	0.35	0.17	0.26	0.86	0.22	0.36	5.19	0.33
h-31	0.12	0.11	0.14	0.23	0.28	0.25	0.44	0.86	0.25	0.29	6.49	0.33
h-32	0.14	0.10	0.16	0.28	0.16	0.30	0.66	1.00	0.36	0.43	6.49	0.37
h-33	0.23	0.16	0.13	0.32	0.23	0.34	0.84	1.36	0.43	0.43	6.49	0.45
h-34	0.43	0.31	0.19	0.36	0.36	0.39	1.46	1.50	0.56	0.71	11.69	0.67
h-35	0.52	0.52	0.34	0.40	0.52	0.45	2.61	1.79	0.72	0.80	23.38	0.95
h-36	0.61	0.40	0.36	0.70	0.38	0.42	1.66	3.21	0.83	1.43	12.99	0.98
h-37	1.23	0.99	0.49	0.99	1.04	0.89	1.29	2.57	1.67	1.66	19.48	1.52
h-38	0.78	0.75	0.47	0.71	0.94	0.78	1.55	2.21	1.64	1.43	27.27	1.37
h-39	0.51	0.60	0.46	0.62	0.70	0.67	1.59	1.57	1.51	1.21	22.08	1.14
h-40	1.0	1.5	0.5	0.8	1.6	0.8	5.1	2.6	0.9	1.4	14.0	1.60
h-41	0.8	1.2	0.7	0.6	1.2	0.7	10.1	3.8	0.8	1.8	17.4	1.68
h-42	1.0	1.4	0.5	0.8	1.5	0.8	2.6	3.9	1.0	2.0	28.6	1.71
h-43	1.4	1.5	0.5	1.2	1.4	0.6	5.1	5.8	0.7	1.3	23.4	1.80
h-44	5.5	0.7	0.4	0.5	0.8	0.3	5.7	3.4	0.4	4.6	17.3	1.53
h-45	0.9	1.7	3.2	2.4	3.1	2.1	47.6	8.9	1.4	36.4	17.5	4.76
h-46	0.9	0.9	0.4	0.7	1.0	0.7	5.5	4.3	0.9	1.6	13.0	1.49
h-47	0.55	0.39	0.35	0.41	0.44	0.52	2.18	7.00	1.21	0.70	28.57	1.09
h-48	0.54	0.49	0.34	0.36	0.58	0.55	1.88	4.50	1.36	0.71	32.47	1.10
h-49	1.13	1.19	0.64	0.82	1.12	0.71	6.14	3.43	1.45	2.21	4.03	1.58
h-50	0.98	1.07	0.66	0.70	0.92	0.67	5.74	3.64	1.40	2.14	5.19	1.52
h-51	1.02	1.01	0.63	0.84	1.22	0.70	5.93	3.86	1.39	2.21	4.55	1.58
h-52	0.74	0.61	0.50	0.46	0.98	0.65	3.17	5.14	1.23	1.50	23.38	1.42
h-53	0.18	0.20	0.37	0.16	0.33	0.55	0.23	0.71	1.48	0.71	1.30	0.43
h-54	0.28	0.23	0.41	0.20	0.37	0.61	0.29	0.79	1.64	0.71	3.90	0.54
h-55	0.41	0.30	0.48	0.27	0.51	0.66	0.38	1.29	1.74	1.07	10.39	0.76
h-56	0.53	0.39	0.50	0.31	0.60	0.68	0.61	1.50	1.90	1.36	12.99	0.92
h-57	0.59	0.62	0.52	0.45	0.73	0.88	0.84	1.79	2.02	1.71	15.58	1.15
h-58	0.94	0.93	0.65	0.61	0.97	0.93	0.98	1.21	2.14	1.07	12.99	1.24
h-59	2.10	0.87	0.70	0.89	1.32	2.18	1.16	2.43	3.60	0.76	16.49	1.73
h-60	1.48	1.09	0.60	0.76	0.90	1.15	1.16	1.81	4.26	1.39	15.32	1.57
h-61	1.07	0.94	0.68	0.82	1.11	0.84	1.03	1.64	2.26	1.64	15.58	1.43
h-62	2.10	0.87	0.70	0.89	1.32	2.18	1.16	2.43	3.60	0.76	16.49	1.73
h-63	1.48	1.05	0.90	0.95	0.96	1.19	0.77	1.37	2.18	1.50	13.90	1.48
h-64	0.82	0.84	0.51	0.48	0.63	0.61	1.16	1.05	2.38	1.25	12.60	1.10
h-65	1.18	0.96	0.66	0.84	1.00	1.20	1.09	1.48	2.78	1.10	13.90	1.43
h-66	0.99	0.91	0.63	0.72	0.72	0.99	0.87	1.29	2.96	1.50	16.88	1.34
h-67	1.02	0.98	0.77	0.97	0.95	0.76	0.81	1.07	2.42	1.43	12.99	1.32
h-68	1.14	1.08	0.71	0.95	1.00	1.15	1.11	1.54	2.40	1.36	19.48	1.52
h-69	1.14	1.02	0.72	0.84	1.04	1.24	1.52	1.54	2.08	1.48	13.90	1.51
h-70	0.82	1.07	0.71	0.82	1.02	1.18	1.19	2.43	2.56	1.71	20.78	1.59
h-71	0.89	1.00	0.68	0.68	0.71	0.85	0.74	0.79	2.40	1.29	12.99	1.17
h-72	0.93	0.95	0.66	0.62	0.63	0.78	0.51	0.93	2.52	1.43	10.39	1.11
h-73	0.86	0.76	0.59	0.51	0.65	0.90	0.64	1.64	2.68	1.64	10.39	1.17
h-74	0.78	0.87	0.53	0.46	0.55	0.62	0.80	0.89	2.02	0.93	12.21	0.99
h-75	1.18	0.96	0.51	0.43	0.49	0.72	0.49	1.00	2.32	1.21	12.99	1.04

h-76	1.52	0.86	0.49	0.70	0.77	1.05	0.84	1.79	2.68	2.00	18.18	1.43
h-77	2.4	1.3	0.8	1.0	1.4	1.5	4.7	4.4	1.9	1.6	15.3	2.14
h-78	2.3	1.2	0.7	1.2	1.5	1.5	5.2	4.9	2.0	1.7	15.1	2.25
h-79	1.3	1.3	0.5	1.0	1.3	1.5	1.6	2.2	2.2	1.5	14.3	1.65
h-80	0.78	0.87	0.41	0.56	0.73	0.65	1.00	3.71	1.68	1.02	13.12	1.18
h-81	1.24	1.04	0.50	0.43	0.64	0.95	1.19	2.21	1.92	1.64	9.09	1.26
h-82	1.51	0.73	0.51	0.38	0.55	0.78	0.86	1.43	2.18	1.29	6.49	1.07
h-83	0.88	0.68	0.51	0.34	0.50	0.72	0.74	1.57	2.30	1.21	6.49	0.98
h-84	1.00	0.61	0.42	0.32	0.49	0.65	0.61	1.21	2.54	1.07	7.79	0.92
h-85	0.75	0.55	0.30	0.39	0.47	0.58	0.54	1.09	0.82	0.76	2.73	0.67
h-86	0.62	0.52	0.33	0.29	0.38	0.49	0.46	1.00	0.97	0.79	2.60	0.62
h-87	0.52	0.49	0.33	0.37	0.46	0.45	0.56	0.76	1.21	0.76	2.36	0.63
h-88	1.09	0.81	0.43	0.53	0.63	1.08	0.59	2.86	2.30	1.64	9.09	1.21
h-89	0.78	0.76	0.46	0.66	0.77	0.91	0.76	1.86	2.64	2.00	10.39	1.23
h-90	0.91	1.22	0.59	0.86	1.36	0.83	1.27	1.71	1.06	1.21	6.49	1.25
h-91	1.07	1.08	0.55	0.62	1.12	1.03	1.02	1.14	1.00	0.71	2.60	1.00
h-92	0.79	1.15	0.39	0.44	1.22	0.92	1.25	1.64	0.94	0.50	2.60	0.93
h-93	1.09	0.91	0.46	0.65	0.93	1.06	1.19	2.21	2.14	1.50	6.49	1.29
h-94	0.91	2.36	0.26	0.81	1.88	0.58	0.87	1.01	0.76	1.00	3.08	1.00
h-95	0.87	1.58	0.31	0.58	1.42	0.64	1.29	1.61	0.51	0.84	4.68	0.99
h-96	0.38	0.71	0.17	0.34	0.94	0.32	3.00	5.36	0.32	0.79	2.36	0.75
h-97	1.00	1.56	0.51	0.95	1.82	0.97	2.29	4.07	0.89	1.52	6.49	1.53
h-98	1.16	2.35	0.35	0.84	2.90	0.74	1.55	22.14	0.69	0.97	3.82	1.58
h-99	0.51	0.84	0.37	1.00	1.02	0.54	0.94	1.43	0.85	1.46	3.60	0.94
h-100	0.48	0.69	0.38	0.93	0.88	0.49	0.62	0.93	0.82	1.14	2.60	0.79
h-101	0.18	0.22	0.19	0.39	0.38	0.32	0.16	0.57	0.63	0.36	1.30	0.35
h-102	0.28	0.34	0.22	0.63	0.48	0.52	0.29	0.93	0.77	0.29	1.30	0.47
h-103	0.4	0.4	0.5	0.5	1.4	0.7	2.1	2.5	0.8	0.7	10.4	1.01
h-104	0.03	0.02	0.15	0.07	0.56	0.15	0.11	0.29	0.38	0.14	1.30	0.15
h-105	0.14	0.06	0.20	0.15	1.06	0.30	0.26	0.64	0.50	0.14	1.30	0.29
h-106	0.32	0.22	0.33	0.36	1.54	0.51	0.98	2.93	0.62	1.00	3.90	0.76
h-107	0.69	1.09	0.66	0.59	2.62	0.97	6.93	23.79	0.69	2.71	6.49	1.94
h-108	0.8	1.7	1.3	0.9	2.0	1.4	54.7	50.7	0.8	3.3	10.6	3.19
h-109	0.84	1.28	0.72	0.66	2.24	1.42	1.91	1.07	1.96	2.36	12.99	1.62
h-110	1.2	1.4	0.6	0.5	2.0	1.6	3.9	4.0	2.1	1.9	23.4	2.05
h-111	0.90	1.20	0.62	0.62	1.86	1.36	1.56	2.71	2.18	1.50	12.99	1.61
h-112	0.81	1.02	0.60	0.50	1.54	1.08	1.00	1.64	2.46	1.07	6.49	1.25
h-113	0.76	0.86	0.55	0.47	1.43	0.98	0.87	1.43	2.34	1.07	6.49	1.15
h-114	0.98	0.85	0.47	0.53	0.93	1.04	1.12	1.79	2.26	1.36	9.09	1.24
h-115	0.71	0.69	0.42	0.43	0.56	0.81	0.69	2.54	2.08	0.95	5.58	0.98
h-116	0.61	0.56	0.38	0.39	0.63	0.73	0.71	2.14	2.38	0.71	3.90	0.88

Table S2. The values of geoaccumulation index of soils (i) and sediments (h). Anthropogenic exposed soils and sediments are marked in red.

Sampling Sites	Igeo (V)	Igeo (Cr)	Igeo (Mn)	Igeo (Co)	Igeo (Ni)	Igeo (Zn)	Igeo (As)	Igeo (Sb)	Igeo (Ba)	Igeo (W)	Igeo (Hg)
					Soils						
i-01	-1.30	-1.79	-1.24	-1.69	-1.16	-0.77	1.65	2.58	-1.98	-0.46	-0.30
i-02	-0.63	-0.57	-1.30	-1.19	-0.65	-0.07	1.77	5.17	-0.79	-0.08	1.97
i-03	-0.92	-1.09	-1.17	-1.60	-0.33	-0.21	1.35	4.56	-0.93	-0.51	2.18
i-04	-0.79	-1.18	-1.02	-2.19	-0.16	-0.52	1.89	3.98	-1.14	-0.93	1.85
i-05	-1.04	-0.82	-1.22	-1.82	-0.55	-0.08	1.56	4.30	-1.29	-1.03	1.49
i-06	-0.11	-0.77	-1.13	-1.46	0.73	0.12	2.29	8.92	-0.87	-0.66	6.42
i-07	-0.48	-0.35	-0.99	-1.25	0.37	0.29	2.02	10.05	-0.53	-0.25	9.19
i-08	-2.60	-2.65	-1.49	-1.42	-0.49	-0.05	-0.81	0.87	-1.00	-0.66	2.00
i-09	-1.38	-1.94	-1.33	-1.19	-0.15	0.12	1.12	1.32	-1.55	-0.31	0.42
i-10	0.63	-0.60	-0.84	-0.82	0.09	0.33	1.89	3.14	-1.20	0.23	1.15
i-11	0.32	-0.51	-0.87	-0.37	-0.09	0.14	1.39	3.27	-1.40	0.38	0.79
i-12	-0.50	-1.06	-1.03	-0.77	-0.78	-0.63	0.87	2.84	-0.92	-0.33	-0.47
i-13	0.18	-0.30	-1.28	-0.30	-0.20	-0.08	1.63	3.00	-1.27	0.30	2.00
i-14	-0.27	-0.39	-1.14	-0.26	-0.06	0.51	1.94	5.30	-0.73	0.72	2.74
i-15	0.01	-0.57	-0.89	-0.57	-0.52	0.30	2.49	7.06	-0.25	0.30	4.12
i-16	0.70	-0.31	-0.70	-0.46	-0.26	0.08	2.17	4.88	-0.29	0.95	5.62
i-17	0.91	-0.41	-0.86	-0.73	0.20	0.23	2.47	4.38	-0.20	1.03	5.06
i-18	-0.39	0.41	-1.30	-0.11	1.05	-0.46	0.35	1.43	-1.37	-0.55	-0.32
i-19	-1.28	-1.06	-1.38	-1.60	-0.75	-1.01	1.78	4.03	-1.47	-0.55	-0.98
i-20	-1.01	-0.88	-1.10	-1.27	-0.47	-0.37	5.06	6.54	-1.12	0.97	-0.27
i-21	-0.74	1.60	-1.59	-1.82	1.44	1.33	9.69	12.06	-0.69	3.15	4.22

i-22	-1.51	-2.86	-2.13	-2.04	-1.85	-1.56	-1.04	1.50	-1.76	0.30	4.50
i-23	-1.00	-1.82	-1.65	-1.53	-1.41	-1.13	0.04	2.37	-1.34	-0.13	4.00
i-24	-0.51	-1.41	-1.30	-1.24	-1.01	-0.67	0.96	4.20	-1.14	-0.58	0.42
i-25	0.07	-0.80	-1.08	-0.46	-0.33	-0.18	2.16	3.89	-0.56	0.34	4.87
i-26	-0.34	-0.88	-0.62	-0.99	-0.52	-0.10	1.10	2.56	-1.44	-0.48	1.74
i-27	-0.54	-0.73	-0.60	-0.95	-0.50	-0.38	1.06	2.27	-1.40	-0.61	1.59
i-28	-1.79	-1.30	-1.50	-2.16	-0.26	-1.16	0.37	-1.58	-2.94	-1.13	0.42
i-29	-1.63	-1.44	-1.57	-2.02	-0.41	-1.48	-0.15	-1.00	-2.75	-1.83	3.00
i-30	-2.54	-2.30	-1.67	-1.78	-0.50	-0.98	0.53	-1.58	-2.53	-2.83	2.00
i-31	-2.33	-2.52	-1.58	-1.73	-1.68	-0.70	0.24	-0.26	-2.04	-1.83	1.42
i-32	-1.46	-2.30	-2.01	-1.31	-0.89	-0.54	1.17	0.87	-2.35	-1.66	1.42
i-33	-0.74	-2.02	-1.42	-0.98	-1.19	-0.31	1.94	1.50	-1.98	-1.13	3.00
i-34	-0.68	-1.55	-1.55	-1.65	-0.52	0.43	1.35	2.50	-1.81	-0.89	1.51
i-35	-0.10	-1.39	-1.08	-0.55	-1.10	0.02	2.29	2.17	-1.29	-0.37	3.42
i-36	0.93	-0.21	-0.58	0.13	0.27	0.87	1.74	3.25	0.08	0.59	5.62
i-37	0.38	-0.52	-0.67	-0.38	0.45	0.40	2.21	2.91	-0.04	0.30	5.54
i-38	0.30	-0.90	-0.76	-0.68	0.13	0.34	2.86	2.77	-0.62	-0.08	4.94
i-39	-0.18	-0.31	-0.76	-0.67	-0.16	0.16	3.51	3.72	-0.95	0.03	1.82
i-40	0.10	0.37	-0.48	0.13	0.22	0.72	5.33	4.51	-0.27	0.07	5.06
i-41	-0.29	-0.57	-0.81	-0.70	-0.14	-0.44	3.40	3.95	-0.89	0.56	-0.25
i-42	0.94	-0.75	0.34	0.37	0.57	1.01	3.78	4.78	-0.73	0.07	-0.08
i-43	-1.38	-1.46	-1.77	-1.55	-0.66	-1.29	1.00	2.69	-1.83	0.66	-0.61
i-44	0.03	-0.21	-0.61	-0.31	0.38	0.09	2.32	2.94	-0.74	0.42	-0.30
i-45	-0.11	-0.60	-0.91	-0.36	0.55	-0.24	3.02	2.58	-1.58	1.38	2.00
i-46	-0.57	-0.88	-1.01	-1.21	-0.31	-0.31	3.21	3.60	-1.48	-0.13	5.22
i-47	-0.29	-0.80	-1.10	-0.89	-0.01	-0.07	3.09	3.84	-1.15	-0.75	5.58
i-48	-0.20	-0.29	-0.55	-0.42	0.16	-0.16	5.06	4.25	-0.48	1.30	-0.67
i-49	-0.60	-0.57	-0.51	-0.75	0.05	0.22	4.32	3.84	-0.83	0.42	0.09
i-50	-0.22	-0.98	-0.95	-1.15	-0.14	0.46	2.12	3.46	-0.56	-0.44	-0.87
i-51	-0.34	-0.54	-0.88	-0.58	-0.10	-0.07	1.11	3.06	-0.57	-0.19	-0.25
i-52	-0.14	-0.94	-0.74	-0.68	0.24	0.08	3.41	3.95	-0.65	0.42	5.00
i-53	-3.45	-2.25	-1.13	-2.04	-1.23	-0.04	-0.96	1.22	-0.26	-0.44	2.74
i-54	-2.94	-2.06	-0.93	-1.78	-0.75	0.10	-1.53	1.12	-0.02	-0.31	2.74
i-55	-2.60	-1.82	-0.82	-1.50	-0.36	0.23	-1.22	2.46	0.02	-0.66	3.00
i-56	-0.63	-1.55	-0.78	-1.19	-0.26	0.38	-0.78	2.12	0.08	-0.31	3.22
i-57	-0.47	-0.86	-0.58	-0.90	-0.10	0.81	-0.15	2.62	0.19	-0.03	3.00
i-58	0.01	-0.57	-0.45	-0.59	0.01	0.12	0.06	2.00	0.38	-0.58	3.42
i-59	0.23	-0.29	-0.38	-0.34	0.15	0.66	0.52	2.66	0.46	-0.03	4.12
i-60	0.19	-0.39	-0.45	-0.42	-0.55	0.52	1.74	2.49	1.23	-0.37	1.62
i-61	0.36	-0.15	-0.41	-0.25	0.30	0.35	0.37	2.87	0.82	-0.19	3.22
i-62	-0.33	-0.65	-0.50	-0.82	-0.85	0.50	0.98	2.39	0.67	-0.75	1.38
i-63	0.04	-0.41	-0.37	-0.44	-0.41	0.81	0.76	2.06	0.59	-0.39	1.52
i-64	-0.29	-0.72	-0.77	-0.97	-0.89	0.29	1.06	2.14	0.41	-0.75	1.43
i-65	0.15	-0.51	-0.34	-0.54	-0.38	0.55	1.54	2.27	0.57	-0.62	1.88
i-66	-0.14	-0.21	-0.26	-0.36	-0.03	0.77	-0.34	2.00	0.69	-0.31	3.00
i-67	0.38	-0.49	-0.25	-0.24	-0.12	-0.46	0.29	1.66	0.53	-0.37	2.42
i-68	0.02	-0.54	-0.61	-0.23	-0.44	0.54	1.30	2.46	0.44	-0.48	1.95
i-69	0.00	-0.35	-0.40	-0.54	-0.41	0.68	1.85	2.46	0.32	-0.54	2.05
i-70	0.59	-0.20	-0.08	-0.15	0.32	0.90	1.42	2.12	0.55	-0.08	3.22
i-71	0.49	-0.45	-0.37	-0.42	-0.44	0.44	0.82	1.32	0.62	-0.31	3.42
i-72	0.66	-0.35	-0.41	-0.53	-0.47	0.26	-0.58	1.32	0.67	-0.19	3.22
i-73	0.52	-0.67	-0.50	-0.69	-0.26	0.55	0.89	2.06	0.77	-0.08	3.00
i-74	0.27	-0.60	-0.46	-1.03	-0.93	0.10	0.10	1.58	0.28	-0.75	4.00
i-75	0.38	-0.49	-0.58	-0.62	-1.01	0.16	-0.68	2.06	0.41	-0.58	3.87
i-76	0.98	-0.29	-0.55	-0.34	0.01	0.88	0.17	3.25	0.78	-0.25	4.00
i-77	0.08	-0.33	0.16	0.94	0.80	1.19	2.28	3.72	-0.08	1.00	-0.04
i-78	0.39	-0.68	-0.05	0.08	0.37	0.68	3.08	4.80	-0.08	0.10	0.07
i-79	6.64	-0.63	-0.71	-0.42	-0.12	0.22	1.76	3.30	0.18	0.03	-0.10
i-80	-0.39	-1.44	-2.54	-0.65	-0.19	0.90	0.12	2.29	-0.44	-0.51	-0.50
i-81	0.56	-1.09	-1.73	-1.29	-0.38	0.94	0.35	2.66	0.17	-0.37	2.74
i-82	0.76	-1.00	-1.37	-1.35	-0.75	0.60	-0.39	2.91	0.26	-0.19	2.42
i-83	0.60	-0.92	-1.24	-1.41	-0.82	0.16	-0.50	2.17	0.32	-0.58	2.74
i-84	0.72	-1.00	-0.78	-1.16	-0.73	0.04	-1.04	2.58	0.56	-0.66	2.42
i-85	-0.51	-1.09	-0.99	-0.97	-0.58	0.29	1.43	3.39	-0.86	-0.06	0.71
i-86	-1.35	-0.90	-0.92	-1.46	-1.10	-0.21	-0.92	3.00	0.07	-1.03	0.42
i-87	-0.21	-0.65	-0.84	-0.87	-0.28	-0.04	0.56	2.17	1.16	-0.19	-0.27
i-88	0.91	-0.58	-0.75	-1.05	-0.50	0.83	0.68	2.46	0.72	-0.19	2.42
i-89	0.07	-0.67	-0.70	-0.52	-0.19	0.59	0.89	2.81	0.84	0.34	2.74
i-90	-1.46	-1.72	-1.15	-1.67	-1.16	-0.24	2.58	1.69	-1.54	-0.83	-0.71
i-91	0.18	-1.06	-1.19	-2.09	-1.62	0.16	1.85	2.12	-1.07	-0.66	0.42

i-92	-0.11	-0.98	-1.10	-1.82	-0.95	-0.11	2.12	1.50	-0.48	-1.37	0.42
i-93	0.31	-0.57	-0.81	-0.54	0.06	0.82	1.77	2.27	0.38	-0.75	2.42
i-94	-0.20	-0.25	-1.68	-0.78	-0.97	0.29	-1.34	1.54	-0.94	-0.45	0.94
i-95	-0.18	0.22	-1.25	-0.42	0.78	-0.26	0.69	2.61	-0.87	-0.41	-0.16
i-96	-0.98	-0.80	-1.88	-1.28	0.05	-0.52	1.75	3.95	-1.60	-0.93	0.42
i-97	0.19	0.32	-1.60	-0.99	0.32	-0.08	1.31	3.81	-0.73	-0.19	3.22
i-98	0.38	0.50	-1.44	-1.15	0.74	-0.38	0.99	4.97	-1.27	-0.66	2.74
i-99	-0.62	-0.72	-1.50	-0.73	0.16	0.18	0.54	3.58	-0.74	-0.25	2.42
i-100	-0.71	-0.96	-1.35	-1.02	-0.05	-0.26	-0.34	3.03	-0.61	-0.58	2.74
i-101	-1.54	-1.62	-1.56	-0.53	-0.38	-0.14	-0.19	1.81	-0.69	-1.25	2.74
i-102	-1.66	-0.84	-1.90	-0.36	-0.36	0.12	0.85	2.06	-0.86	-1.13	4.12
i-103	-0.68	0.22	-0.58	-0.97	0.38	0.47	3.32	3.14	-0.31	-0.75	5.54
i-104	-4.75	-5.00	-1.67	-3.53	0.15	-0.98	-1.92	1.00	-1.43	-2.51	2.74
i-105	-2.48	-2.86	-1.40	-2.11	0.49	-0.46	-0.29	2.06	-1.08	-2.51	4.12
i-106	-0.88	-1.11	-1.06	-1.12	0.76	0.14	1.62	4.09	-0.74	-0.58	4.66
i-107	-0.38	-0.35	-0.83	-1.82	0.45	0.09	2.59	5.23	-1.10	-0.13	2.42
i-108	0.19	0.71	-0.39	-0.92	-0.10	1.36	5.29	7.86	-0.92	0.38	2.74
i-109	0.72	-0.05	-0.61	-0.87	0.34	1.10	1.64	4.26	0.41	0.34	3.00
i-110	0.98	0.87	-0.82	0.10	0.89	1.54	4.19	5.42	0.33	0.72	4.94
i-111	0.35	0.59	-0.77	-0.70	0.73	1.30	3.11	3.68	0.55	-0.31	4.00
i-112	-0.24	-0.10	-0.55	-0.94	-0.41	1.10	2.20	3.25	0.46	-0.37	3.22
i-113	-0.30	-0.49	-0.45	-0.78	-0.26	0.64	1.91	2.87	0.64	-0.66	2.74
i-114	0.06	-0.67	-0.75	-1.06	-0.28	-0.04	1.44	3.44	0.37	0.38	3.42
i-115	-1.12	-1.18	-1.31	-1.64	-1.01	-0.67	0.92	5.18	-0.06	1.40	-0.56
i-116	-0.66	-1.11	-1.18	-1.31	-0.75	0.23	0.77	4.16	0.19	0.75	1.42
Sediment											
h-01	-0.91	-0.78	-1.80	-1.25	-1.02	-1.04	0.49	2.15	-0.35	0.11	1.98
h-02	-2.72	-2.64	-2.20	-1.87	-1.64	-2.16	0.78	1.19	-2.07	-0.45	0.58
h-03	-1.12	-0.77	-2.16	-1.35	-1.08	-0.80	-0.21	2.76	-0.15	0.04	2.11
h-04	-1.08	-0.69	-2.18	-1.20	-1.02	-0.61	-0.71	3.43	-0.08	0.37	3.61
h-05	-0.98	-0.62	-2.27	-1.40	-0.62	-1.12	-1.04	3.89	-0.12	0.61	3.94
h-06	-1.02	-0.55	-2.58	-0.85	-0.29	-0.57	-1.21	4.65	-0.04	0.69	4.94
h-07	-0.62	-0.38	-2.46	-0.07	0.08	-0.41	-0.15	6.41	0.08	1.03	8.30
h-08	-0.92	-0.47	-2.32	-0.66	0.51	-0.27	-3.45	11.36	0.10	1.39	10.24
h-09	-2.07	-1.87	-1.82	-2.43	-1.66	-1.50	-1.52	0.13	-0.29	-1.58	2.11
h-10	-2.21	-1.64	-1.76	-2.11	-1.30	-1.42	-0.88	1.00	-0.56	-0.93	2.11
h-11	-0.95	-1.02	-1.66	-1.19	-1.14	-0.93	0.00	2.80	-0.49	-0.05	2.11
h-12	-1.03	-0.91	-1.75	-1.40	-1.37	-1.08	0.09	2.29	-0.55	-0.22	2.60
h-13	-0.43	-0.36	-1.57	-0.55	-0.53	-0.50	1.39	1.31	-0.29	0.56	3.66
h-14	-0.81	-0.62	-2.03	-1.22	-0.98	-0.92	0.62	1.70	-0.35	0.42	2.60
h-15	-0.98	-0.78	-1.81	-1.48	-0.86	-1.52	0.37	1.78	-0.59	0.25	2.79
h-16	-1.17	-0.97	-1.76	-1.08	-1.08	-1.30	0.11	2.07	-0.60	-0.21	1.06
h-17	-0.97	-0.80	-1.73	-1.31	-1.14	-1.48	0.00	2.63	-0.53	-0.14	0.95
h-18	-0.49	-0.89	-1.62	-1.25	-0.24	-1.25	0.47	3.57	-0.45	0.82	3.96
h-19	-0.97	-0.95	-1.17	-0.97	-1.12	-0.79	-0.77	-1.63	0.09	-0.81	1.90
h-20	-0.89	-1.27	-1.42	-1.17	-0.61	-1.18	-0.16	-0.58	-0.18	-0.63	3.11
h-21	-1.40	-0.82	-1.78	-1.55	-1.08	-1.65	1.25	-0.37	-0.38	-0.21	4.15
h-22	-1.61	-0.08	-2.96	-2.01	-1.08	0.13	7.32	7.91	-1.52	3.74	6.35
h-23	-2.54	-3.21	-3.40	-3.63	-3.16	-3.44	-1.25	-1.81	-1.94	-1.07	3.11
h-24	-1.91	-2.24	-2.92	-2.48	-2.30	-2.75	-0.91	-1.39	-1.80	-0.81	3.38
h-25	-1.79	-1.82	-2.34	-2.01	-1.84	-1.99	-0.33	-0.02	-1.02	-0.81	3.76
h-26	-0.76	-1.06	-2.10	-1.63	-2.73	-2.25	0.25	2.53	-0.62	-1.07	3.70
h-27	-1.88	-1.88	-2.98	-2.63	-1.58	-1.18	-1.91	-0.46	-1.78	-1.06	5.04
h-28	-2.43	-1.98	-3.62	-3.14	-2.42	-3.17	-2.22	-0.52	-3.21	-0.96	2.42
h-29	-2.97	-2.35	-3.66	-2.87	-2.55	-3.08	-2.36	-1.39	-2.96	-2.07	1.79
h-30	-2.80	-2.72	-3.74	-3.03	-2.12	-3.16	-2.50	-0.81	-2.78	-2.07	1.79
h-31	-3.65	-3.73	-3.43	-2.73	-2.40	-2.56	-1.76	-0.81	-2.61	-2.39	2.11
h-32	-3.43	-3.92	-3.19	-2.43	-3.27	-2.32	-1.18	-0.58	-2.04	-1.81	2.11
h-33	-2.70	-3.26	-3.55	-2.22	-2.68	-2.15	-0.83	-0.14	-1.80	-1.81	2.11
h-34	-1.81	-2.28	-2.98	-2.07	-2.06	-1.93	-0.04	0.00	-1.43	-1.07	2.96
h-35	-1.54	-1.53	-2.14	-1.91	-1.53	-1.72	0.80	0.25	-1.05	-0.91	3.96
h-36	-1.30	-1.92	-2.06	-1.10	-1.98	-1.85	0.15	1.10	-0.86	-0.07	3.11
h-37	-0.28	-0.60	-1.61	-0.60	-0.53	-0.75	-0.22	0.78	0.15	0.15	3.70
h-38	-0.95	-1.01	-1.66	-1.08	-0.67	-0.93	0.05	0.56	0.13	-0.07	4.18
h-39	-1.55	-1.32	-1.71	-1.28	-1.10	-1.16	0.08	0.07	0.01	-0.30	3.88
h-40	-0.63	-0.01	-1.69	-0.83	0.13	-0.88	1.78	0.79	-0.76	-0.14	3.23
h-41	-0.92	-0.36	-1.11	-1.28	-0.35	-1.05	2.76	1.34	-0.96	0.23	3.54
h-42	-0.66	-0.14	-1.50	-0.83	-0.04	-0.89	0.82	1.36	-0.65	0.39	4.25
h-43	-0.06	-0.02	-1.57	-0.37	-0.14	-1.34	1.76	1.95	-1.05	-0.22	3.96

h-44	1.86	-1.14	-1.87	-1.47	-0.98	-2.22	1.93	1.16	-2.09	1.61	3.53
h-45	-0.76	0.21	1.08	0.66	1.04	0.48	4.99	2.57	-0.08	4.60	3.55
h-46	-0.76	-0.69	-1.75	-1.17	-0.64	-1.01	1.87	1.51	-0.74	0.11	3.11
h-47	-1.45	-1.95	-2.10	-1.87	-1.77	-1.52	0.54	2.22	-0.31	-1.10	4.25
h-48	-1.48	-1.62	-2.15	-2.07	-1.37	-1.44	0.32	1.58	-0.14	-1.07	4.44
h-49	-0.42	-0.34	-1.22	-0.87	-0.42	-1.08	2.03	1.19	-0.05	0.56	1.42
h-50	-0.61	-0.49	-1.18	-1.10	-0.71	-1.16	1.94	1.28	-0.10	0.51	1.79
h-51	-0.56	-0.57	-1.24	-0.83	-0.30	-1.10	1.98	1.36	-0.11	0.56	1.60
h-52	-1.02	-1.31	-1.60	-1.70	-0.61	-1.22	1.08	1.78	-0.29	0.00	3.96
h-53	-3.10	-2.91	-2.04	-3.20	-2.18	-1.46	-2.71	-1.07	-0.02	-1.07	-0.21
h-54	-2.45	-2.72	-1.88	-2.91	-2.04	-1.29	-2.39	-0.93	0.13	-1.07	1.38
h-55	-1.88	-2.31	-1.65	-2.45	-1.55	-1.19	-1.99	-0.22	0.21	-0.49	2.79
h-56	-1.51	-1.94	-1.57	-2.27	-1.33	-1.15	-1.30	0.00	0.34	-0.14	3.11
h-57	-1.34	-1.28	-1.54	-1.73	-1.05	-0.76	-0.83	0.25	0.43	0.19	3.38
h-58	-0.67	-0.69	-1.22	-1.31	-0.63	-0.69	-0.62	-0.30	0.51	-0.49	3.11
h-59	0.49	-0.78	-1.09	-0.75	-0.18	0.54	-0.37	0.70	1.26	-0.99	3.46
h-60	-0.02	-0.46	-1.32	-0.98	-0.74	-0.39	-0.37	0.27	1.51	-0.11	3.35
h-61	-0.49	-0.67	-1.14	-0.87	-0.43	-0.84	-0.54	0.13	0.59	0.13	3.38
h-62	0.49	-0.78	-1.09	-0.75	-0.18	0.54	-0.37	0.70	1.26	-0.99	3.46
h-63	-0.02	-0.52	-0.74	-0.66	-0.64	-0.33	-0.96	-0.13	0.54	0.00	3.21
h-64	-0.88	-0.84	-1.57	-1.65	-1.26	-1.30	-0.37	-0.51	0.67	-0.26	3.07
h-65	-0.35	-0.64	-1.18	-0.83	-0.58	-0.32	-0.46	-0.02	0.89	-0.45	3.21
h-66	-0.60	-0.73	-1.25	-1.06	-1.07	-0.60	-0.78	-0.22	0.98	0.00	3.49
h-67	-0.56	-0.62	-0.96	-0.62	-0.65	-0.98	-0.89	-0.49	0.69	-0.07	3.11
h-68	-0.39	-0.47	-1.09	-0.66	-0.58	-0.38	-0.43	0.03	0.68	-0.14	3.70
h-69	-0.39	-0.55	-1.06	-0.83	-0.53	-0.28	0.02	0.03	0.47	-0.02	3.21
h-70	-0.88	-0.49	-1.08	-0.88	-0.55	-0.35	-0.33	0.70	0.77	0.19	3.79
h-71	-0.75	-0.58	-1.14	-1.13	-1.08	-0.83	-1.03	-0.93	0.68	-0.22	3.11
h-72	-0.70	-0.65	-1.18	-1.28	-1.25	-0.95	-1.54	-0.69	0.75	-0.07	2.79
h-73	-0.81	-0.97	-1.36	-1.57	-1.20	-0.74	-1.22	0.13	0.84	0.13	2.79
h-74	-0.94	-0.78	-1.49	-1.70	-1.44	-1.27	-0.91	-0.76	0.43	-0.69	3.02
h-75	-0.35	-0.64	-1.57	-1.81	-1.61	-1.05	-1.63	-0.58	0.63	-0.30	3.11
h-76	0.02	-0.80	-1.62	-1.10	-0.95	-0.52	-0.84	0.25	0.84	0.42	3.60
h-77	0.65	-0.24	-0.96	-0.58	-0.14	-0.02	1.65	1.54	0.35	0.05	3.35
h-78	0.64	-0.27	-1.03	-0.35	0.00	0.04	1.80	1.70	0.40	0.17	3.33
h-79	-0.16	-0.23	-1.69	-0.56	-0.23	0.04	0.07	0.56	0.53	-0.03	3.25
h-80	-0.95	-0.78	-1.87	-1.41	-1.04	-1.20	-0.58	1.31	0.17	-0.55	3.13
h-81	-0.27	-0.53	-1.58	-1.80	-1.22	-0.65	-0.34	0.56	0.36	0.13	2.60
h-82	0.01	-1.04	-1.54	-1.98	-1.44	-0.93	-0.81	-0.07	0.54	-0.22	2.11
h-83	-0.76	-1.14	-1.56	-2.13	-1.58	-1.07	-1.03	0.07	0.62	-0.30	2.11
h-84	-0.58	-1.29	-1.82	-2.25	-1.62	-1.20	-1.30	-0.30	0.76	-0.49	2.38
h-85	-1.00	-1.44	-2.34	-1.95	-1.69	-1.36	-1.47	-0.46	-0.87	-0.97	0.86
h-86	-1.28	-1.53	-2.19	-2.37	-1.98	-1.61	-1.69	-0.58	-0.63	-0.93	0.79
h-87	-1.54	-1.62	-2.17	-2.03	-1.71	-1.72	-1.43	-0.97	-0.31	-0.97	0.66
h-88	-0.46	-0.89	-1.81	-1.51	-1.25	-0.47	-1.36	0.93	0.62	0.13	2.60
h-89	-0.95	-0.97	-1.70	-1.19	-0.96	-0.72	-0.97	0.31	0.82	0.42	2.79
h-90	-0.72	-0.29	-1.35	-0.80	-0.14	-0.85	-0.24	0.19	-0.50	-0.30	2.11
h-91	-0.49	-0.47	-1.45	-1.28	-0.42	-0.54	-0.55	-0.39	-0.58	-1.07	0.79
h-92	-0.92	-0.38	-1.95	-1.78	-0.30	-0.71	-0.26	0.13	-0.67	-1.58	0.79
h-93	-0.46	-0.73	-1.71	-1.21	-0.69	-0.50	-0.34	0.56	0.51	0.00	2.11
h-94	-0.72	0.66	-2.53	-0.90	0.33	-1.36	-0.78	-0.56	-0.98	-0.58	1.04
h-95	-0.79	0.07	-2.26	-1.37	-0.08	-1.23	-0.22	0.10	-1.55	-0.83	1.64
h-96	-2.00	-1.09	-3.18	-2.15	-0.67	-2.22	1.00	1.84	-2.23	-0.93	0.66
h-97	-0.58	0.06	-1.55	-0.66	0.28	-0.63	0.61	1.44	-0.76	0.02	2.11
h-98	-0.37	0.65	-2.09	-0.83	0.95	-1.02	0.05	3.88	-1.11	-0.63	1.35
h-99	-1.56	-0.84	-2.01	-0.58	-0.56	-1.48	-0.67	-0.07	-0.83	-0.03	1.26
h-100	-1.66	-1.11	-1.98	-0.69	-0.77	-1.61	-1.27	-0.69	-0.88	-0.39	0.79
h-101	-3.10	-2.75	-3.00	-1.93	-1.98	-2.22	-3.19	-1.39	-1.25	-2.07	-0.21
h-102	-2.45	-2.14	-2.78	-1.25	-1.64	-1.54	-2.36	-0.69	-0.97	-2.39	-0.21
h-103	-1.94	-1.95	-1.60	-1.58	-0.14	-1.10	0.48	0.74	-0.86	-1.07	2.79
h-104	-5.91	-5.99	-3.30	-4.45	-1.42	-3.29	-3.71	-2.39	-2.00	-3.39	-0.21
h-105	-3.40	-4.67	-2.89	-3.30	-0.50	-2.32	-2.54	-1.22	-1.60	-3.39	-0.21
h-106	-2.24	-2.75	-2.20	-2.05	0.04	-1.56	-0.62	0.97	-1.27	-0.58	1.38
h-107	-1.12	-0.46	-1.18	-1.35	0.80	-0.63	2.21	3.99	-1.12	0.86	2.11
h-108	-0.92	0.20	-0.19	-0.79	0.39	-0.10	5.19	5.08	-0.84	1.13	2.83
h-109	-0.83	-0.23	-1.05	-1.18	0.58	-0.08	0.35	-0.49	0.39	0.65	3.11
h-110	-0.33	-0.05	-1.22	-1.51	0.43	0.07	1.37	1.42	0.49	0.31	3.96
h-111	-0.74	-0.32	-1.28	-1.27	0.31	-0.14	0.06	0.86	0.54	0.00	3.11
h-112	-0.89	-0.55	-1.33	-1.58	0.04	-0.47	-0.58	0.13	0.71	-0.49	2.11
h-113	-0.98	-0.80	-1.45	-1.66	-0.07	-0.61	-0.78	-0.07	0.64	-0.49	2.11

h-114	-0.62	-0.82	-1.66	-1.51	-0.70	-0.53	-0.42	0.25	0.59	-0.14	2.60
h-115	-1.08	-1.11	-1.84	-1.80	-1.42	-0.89	-1.13	0.76	0.47	-0.66	1.90
h-116	-1.30	-1.41	-1.98	-1.93	-1.26	-1.04	-1.07	0.51	0.67	-1.07	1.38
